# Time-Restricted Eating Benefits on Pulmonary Function and Postural Balance in Overweight or Obese Women

**DOI:** 10.3390/nu16172919

**Published:** 2024-09-01

**Authors:** Sarra Miladi, Omar Hammouda, Ranya Ameur, Sirine C. Miladi, Walid Feki, Tarak Driss

**Affiliations:** 1 Interdisciplinary Laboratory in Neurosciences, Physiology, and Psychology: Physical Activity, Health, and Learning—LINP2, UFR STAPS, Paris Nanterre University, 92000 Nanterre, France; m.sarra@parisnanterre.fr (S.M.); omar.hammouda@parisnanterre.fr (O.H.); 2 Research Laboratory, Molecular Bases of Human Pathology, LR19ES13, Faculty of Medicine, University of Sfax, Sfax 3000, Tunisia; 3 High Institute of Sport and Physical Education of Sfax, University of Sfax, Sfax 3000, Tunisia; ranyaamer@gmail.com (R.A.); sirinemiledi376@gmail.com (S.C.M.); 4 Research Laboratory of Evaluation and Management of Musculoskeletal System Pathologies, LR20ES09, University of Sfax, Sfax 3000, Tunisia; 5 Department of Respiratory Medicine, Hedi Chaker Hospital, University of Sfax, Sfax 3000, Tunisia; fki_walid@yahoo.fr

**Keywords:** time-restricted eating, heart rate variability, circadian rhythms, health

## Abstract

This study aimed to evaluate the impact of time-restricted eating (TRE) on neuro-physiological parameters, objective and subjective sleep, pulmonary capacity, and postural balance among women with excess body weight. Methods: Thirty-one participants were assigned to either a TRE group (n = 15, 28.74 ± 9.25 years, 88.32 ± 13.38 kg, and 32.71 ± 5.15 kg/m^2^), engaging in ad libitum 16 h fasting over a 12-week period, or a control group (CG, n = 16, 36.25 ± 11.52 years, 90.88 ± 19.01 kg, and 33.66 ± 6.18 kg/m^2^). The assessment of heart rate variability (HRV), spirometric parameters (forced vital capacity (FVC), forced expiratory volume in the first second (FEV1), FEV1/ FVC ratio, objective and subjective sleep assessments employing actigraphy and the Epworth Sleepiness Scale, and postural balance using the Y balance test (YBT) were conducted before and after the intervention. Results: No significant negative effects of TRE were observed for HRV and objective sleep parameters. Only the TRE group improved FEV1 in both sitting (*p* < 0.0005) and supine positions (*p* = 0.001). Furthermore, the TRE group showed improvement in postural balance performance compared to the CG in anterior (*p* = 0.03), postero-medial (*p* = 0.04), and postero-lateral directions (*p* = 0.003). Conclusion: This study highlights TRE as a feasible and safe dietary intervention with significant improvements in postural balance and pulmonary function, without any negative impact on HRV or objective sleep assessments among overweight or obese women.

## 1. Introduction

Obesity is considered as a global health challenge, linked to various health issues, including cardiometabolic disorders [1], diminished quality of life, and increased risk of premature death [2]. The autonomic nervous system disruption characterized by increased sympathetic activity [3] and decreased parasympathetic function [4] in this population plays a pivotal role in the onset of cardiovascular, respiratory, and metabolic complications in individuals dealing with obesity [5].

The major respiratory complications of obesity include a heightened demand for ventilation, elevated work of breathing, respiratory muscle inefficiency, and diminished respiratory compliance [6]. The decrease of functional residual capacity (FRC) and expiratory reserve volume observed in obese, along with an elevated closing volume to FRC ratio leads to ventilation-perfusion abnormalities and subsequent hypoxemia [7], especially in the supine position. Conventional respiratory function tests are affected by obesity except in extreme cases [8]. The major circulatory complications are increased total and pulmonary blood volume, high cardiac output, and elevated right ventricular and diastolic pressure [9]. Patients with obesity commonly develop hypoventilation and sleep apnea syndromes with attenuated hypoxic and hypercapnic ventilatory responsiveness [10]. The result is pulmonary hypertension, and progressively worsening disability. Obese patients have increased dyspnea [11] and decreased exercise capacity, which are vital to the quality of life [12,13]. Decreased muscle mass, increased joint pain, and skin friction are important determinants of decreased exercise capacity, in addition to the cardiopulmonary effects of obesity [14]. The effects of obesity on mortality in heart failure and chronic obstructive pulmonary disease have not been definitively resolved [15]. Whether obesity contributes to asthma and airway hyper-responsiveness is uncertain [16]. Weight reduction and physical activity are effective means of reversing the respiratory complications of obesity [12]. However, it is important to note that most previous studies have primarily compared obese individuals to those with normal weight and measured FRC, and, to some extent, total lung capacity [17]. Moreover, postural change-induced alterations in lung function are recognized to affect normal lung volumes due to the gravitational effects of abdominal contents on the diaphragm’s position [17,18].

Moreover, the bidirectional relationship between sleep quality and obesity is complex, as sleep disruption can contribute to the high prevalence of conditions such as, hypoglycemia, and nocturnal symptoms like nocturia or painful neuropathy [19].

Time-restricted eating (TRE), a form of intermittent fasting [20], has gained attention as a promising intervention for addressing various health concerns [21], especially in overweight and obese adults [22,23].

Fasting has been linked with some cardiovascular benefits including decreased risk of major cardiac events [24,25]. Most of the previous research have focused on the physiological benefits of TRE, including weight loss and improvements in cardiovascular health [26]. Furthermore, heart rate variability (HRV) is known as a valuable indicator of cardiovascular health and a valid method for estimating cardiac autonomic function. However, limited research investigating the impact of fasting on HRV have shown no definitive conclusion [27]. Kammoun et al. [28] have investigated the effect of Ramadan intermittent fasting (RIF) on HRV in overweight men and reported an alteration of some parameters of the parasympathetic activity (i.e., high frequency (HF) and low frequency (LF)). Cansel et al. [29] have reported higher HRV values after RIF in healthy individuals.

While the relationship between cardiovascular and respiratory dynamics is acknowledged, it prompts additional investigation regarding the potential influence of interventions such as TRE on both cardiac autonomic control and pulmonary functions. This necessitates a thorough evaluation to understand the potential relationship between these physiological processes.

Otherwise, previous findings supported the involvement of the autonomic nervous system in postural control with a higher contribution of the sympathetic system [30]. Achieving postural stability depends on the interaction between the homeostasis of various systems and the integration of multi-tiered inputs and processing within the nervous system. Previous research highlighted the significant influence of sensory, cognitive, and behavioral factors on the motor system, with the state of an individual’s nervous system at the time of movement execution [31].

Throughout the literature, studies have investigated the impact of emotional states, such as stress, often utilizing cortisol levels as a key indicator. Elevated cortisol levels, characteristic of heightened stress, are associated with a decline in executive function and have the potential to affect negatively the postural balance [32]. In this sense, there is little information about the impact of TRE on postural balance in women with obesity. Some previous studies showed that RIF altered postural balance in both athletes [33] and elderly [34]. The postural impairment due to intermittent fasting could be attributed to changes in circadian rhythms, including mealtimes and cumulative sleep deprivation [35]. Obviously, sleep deficit can degrade the vestibular, muscular, and autonomic nervous systems and thus negatively affect the physiological aspects of postural function [36]. From a hormonal point of view, melatonin is recognized as a sleep regulator in humans. Kamoun et al. [37] have shown that the decline of melatonin levels in older adults correlates with cognitive impairments, notably short-term memory deficits, which in turn can precipitate vestibular dysfunction and disturbances in balance. Our study aimed to investigate the effect of TRE on HRV, objective and subjective sleep, pulmonary function, and postural balance in overweight or obese women.

## 2. Materials and Methods

### 2.1. Participants

The minimum required sample size was determined using the G*power software (version 3.1.9.6; Kiel University, Kiel, Germany). Based on prior research [38], the α and power (1 − β) values were set at 0.05 and 0.8, respectively. To ensure sufficient statistical power and minimize the risk of a type 2 error, data from twenty-four participants were deemed necessary. 

**Figure 1:**
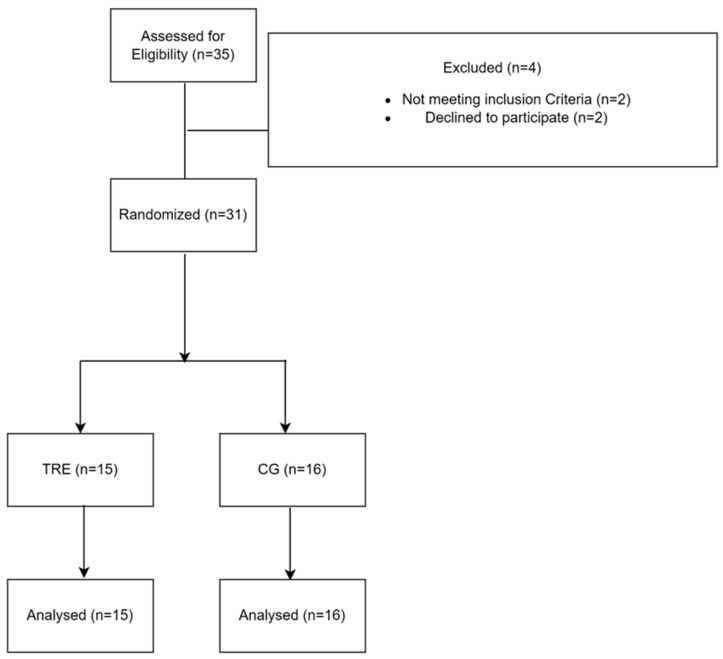
Flow chart of participants’ recruitment. TRE: time-restricted eating group; CG: control group.

Thirty-one overweight or obese women were enrolled in the present study (Figure 1). The assessments of weight and body composition were conducted using a bioelectrical impedance technique (Model TBF-300, Tanita corp, Tokyo, Japan). Besides, energy expenditure (EE) and the metabolic equivalent of task (METs/h) were measured using the Actigraph GT9X (ActiGraph Corp, Pensacola, FL, USA) to observe the evolution of the participants throughout the study. Prior to their inclusion, the participants were informed about the details of the experimental protocol and the research objectives and signed an informed consent. They also underwent a thorough medical examination and completed the Short Form Health Survey Questionnaire SF-36 [39]. This process aimed to evaluate the participant’s quality of life and to confirm the absence of regular medication use, a history of cardiopulmonary or neurological diseases, and established non-smoking status. Furthermore, the participants with a BMI > 25 kg/m^2^ were included, while those with any physical injuries or mobility concerns that could interfere with the study’s objectives were excluded. Additionally, the participants had abstained from regular physical activity and any diet or fasting programs in the six months preceding the study. The study design received formal approval from the local ethics committee (CPP SUD, n ° 0460/2022, Sfax, Tunisia) and was registered in the Pan African Clinical Trials Registry (PACTR202301473691345) and complied with Helsinki’s declaration for human experimentation.

### 2.2. Study Design

The study lasted 14 weeks, during which the participants were randomly allocated to either the time-restricted eating group (TRE, n= 15, 28.74 ± 9.25 years, 88.32 ± 13.38 Kg, and 32.71 ± 5.15 Kg/m^2^) or control group (CG, n= 16, 36.25 ± 11.52, 90.88 ± 19.01 Kg, and 33.66 ± 6.18 Kg/m^2^). The assessments of HRV, spirometric measures (forced vital capacity (FVC)), (forced expiratory volume in the first second (FEV1)) and (FEV1/FVC), Y balance test (YBT) in three different directions (anterior [A], postero-medial [PM], and postero-lateral [PL] with both legs, and subjective and objective sleep patterns were conducted before and after the intervention. 

#### 2.2.1. Time-Restricted Eating Diet

**Figure 2:**
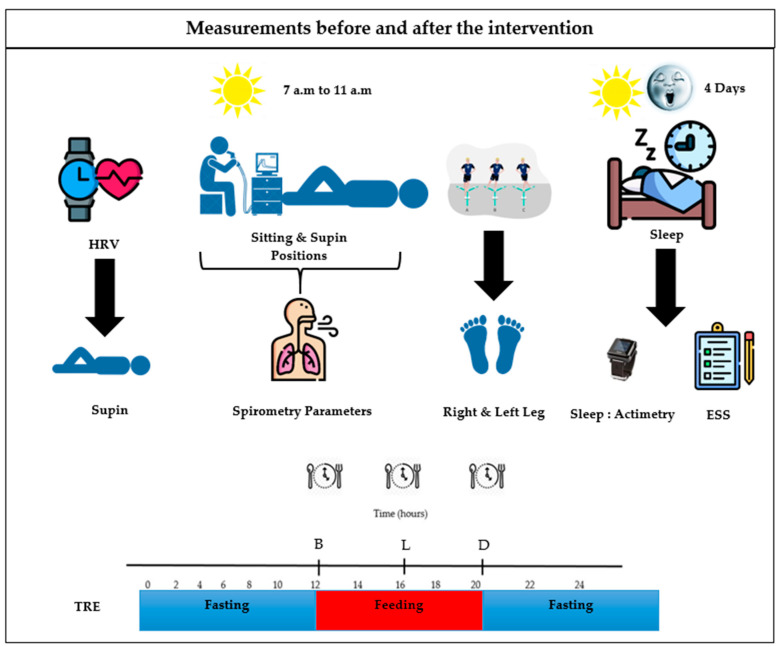
Dietary guidelines intervention. TRE: time-restricted eating group. B: breakfast; L: lunch; D: dinner.

Ad libitum approach without caloric restriction was employed to explore the impact of eating patterns. The participants of the TRE group were instructed to fast between 8:00 p.m. and 12:00 p.m. establishing a 16 h fasting window (Figure 2), during which only water, tea, and coffee were allowed. The selection of the eating window was based on the established literature on TRE [24,40,41], while also considering the participants preferences. Furthermore, the participants received personalized recommendations regarding both the frequency of meals and calorie intake to optimize the health outcomes of the intervention [41]. Conversely, the CG participants were instructed to maintain their regular eating routines.

#### 2.2.2. Control Condition

Throughout the study, the participants in the CG were instructed to adhere to their regular daily meal and sleep routines without including any fasting protocols. To ensure methodological consistency, all the measurements were taken at the same time of day.

### 2.3. HRV Analysis

The RR intervals were recorded using the Polar technology (Polar V800, Polar Electro OY, Kemplete, Finland). The data collection window was set between 8:00 and 10:30 a.m. to mitigate the potential circadian influences on cardiac autonomic function. Before the measurement, the participants assumed a supine position for a minimum of 10 min to ensure a stable baseline. During the HRV recording, the participants were given specific instructions to lie, close their eyes, refrain from verbal communication, and maintain a normal breathing frequency. To neutralize the impact of respiratory sinus arrhythmia on HRV, the respiratory frequency was maintained at a constant rate of 0.25 Hz, achieved by synchronizing recorded breathing sounds. Before the signal analysis, all ectopic beats were filtered and corrected. The HRV analysis included several parameters in the time domain: the mean of the R-R intervals, the standard deviation of the normal R-R interval (SDNN), the root mean square of the successive differences in the R-R intervals (RMSSD), and the percentage of successive interval differences larger than 50 ms (PNN50). Frequency-domain parameters were derived through fast Fourier transformation, quantifying the low-frequency bands (LF) (0.04–0.15 Hz), high frequency (HF) (0.15–0.40 Hz), and the LF/HF ratio expressed in normalized units (nu). Consistency in data analysis was maintained with the same researcher employing the premium Kubios HRV software (v. 3.1.1.).

### 2.4. Sleep Patterns

The objective sleep measurements were obtained using tri-axial accelometers (Actigraph GT9X, ActiGraph Corp, Pensacola, FL, USA). The data were collected continuously over a four-day period during each testing session, comprising three weekdays and one weekend day. Non-wearing time was defined as periods of 60 min or more without activity counts [42]. The following sleep parameters were extracted using the actilife software (v.6.13.7; Actigraph LLC, Pensacola, FL, USA): total sleep time (TST), time in bed (TIB), sleep efficiency (SE), sleep onset latency (SL), and wake after sleep onset (WASO). Moreover, all the participants underwent the subjective Epworth Sleepiness Scale (ESS) to assess an overall level of daytime sleepiness. The ESS scores were calculated following the instructions provided by Tinsely [43].

### 2.5. Respiratory Function Measurements 

The spirometry evaluations were conducted between 08:00 and 10:30 a.m. according to the guidelines presented by Miller [44]. The tests were conducted in both sitting and supine positions. The Mir Spirobank II Smart (Medical International Research, Rome, Italy) was used to assess respiratory functions, employing FVC maneuvers. The tests were consistently conducted in a controlled environment utilizing the same room and operator. The participants were instructed to perform the test while maintaining an upright-seated position with their neck and chest aligned. After 15 min rest break the participants repeated the test in a supine position. The largest FVC, FEV1, and FEV1/FVC were selected to represent the results for each patient.

### 2.6. Y Balance Test (YBT)

The Y balance test (YBT) was conducted to assess the participants’ postural balance. Procedures were used as detailed in a previous study [45]. The participants were asked to stand on one leg while extending the opposite limb in three distinct directions: [A], [PM], and [PL]. The extension was performed using the great toe of their testing leg, reaching as far as possible. To familiarize themselves with the test, the participants underwent three practice trials in each direction with each leg. During the actual test, the participants were given clear instructions to maintain their testing leg centered on the grid, facing forward with hands on their hips throughout the trial. They were required to return to the starting position after each attempt.

### 2.7. Statistical Analysis

The data analysis process was conducted with SPSS version 25 for Windows (SPSS Inc., Chicago, II, USA). The values are presented as mean ± standard deviation (SD). To assess the normality of the data, the Kolmogorov/Smirnov test was employed. To analyze the spirometric parameters (FVC, FEV1, and FEV1/FVC) a three-way ANOVA for repeated measures was applied to evaluate the differences across (2 groups (TRE and CG) × 2 measurement points (before and after the intervention) × 2 positions (sitting and supine)). Similarly, for the YBT, a three-way ANOVA for repeated measures was applied to evaluate differences across (2 groups (TRE and CG) × 2 measurement points (before and after the intervention) × 2 legs used (right and left)) across three directions (anterior [A], postero-medial [PM], and postero-lateral [PL]). The data of the sleep measurements, body composition, energy expenditure, and metabolic equivalent of task (MET/s) were analyzed using a two-way ANOVA for repeated measures (2 groups (TRE and CG) × 2 measures point (before and after the intervention)). To assess the practical significance of the findings, effect sizes were computed using partial eta-squared (ηp^2^) for the ANOVA. These effect sizes were classified according to Lakens [46], with values of 0.01, 0.06, and 0.13 indicating small, moderate, and large effect sizes, respectively. When significant main or interaction effects were observed, pairwise comparisons were performed using the Bonferroni post hoc test. The HRV was analyzed using the Mann/Whitney non-parametric test, with a calculation between post- and pre-intervention values (∆). 

## 3. Results

### 3.1. Participants Characteristics

The participants’ characteristics are presented in Table 1. The Two-way repeated measures ANOVA showed significant effects of time and interaction time × groups for weight (Table 1). However, only a time × groups interaction effect was observed for FFM and MET/s (Table 1). The Bonferroni post hoc test showed a higher reduction in weight in the TRE group after the intervention compared to before the intervention (*p* < 0.0005). Furthermore, a higher enhancement in MET/s was observed in the TRE group compared to the CG group (*p* = 0.003). 

### 3.2. HRV

#### 3.2.1. Time Domain

The statistical analysis revealed non-significant changes (i.e., mean HR, mean RR, SDNN, RMSSD, and PNN50 in both groups after the intervention.

#### 3.2.2. Frequency Domain

The statistical analysis revealed non-significant changes (i.e., HF, LF, and LF/HF) after the intervention. 

### 3.3. Sleep Parameters

**Figure 3:**
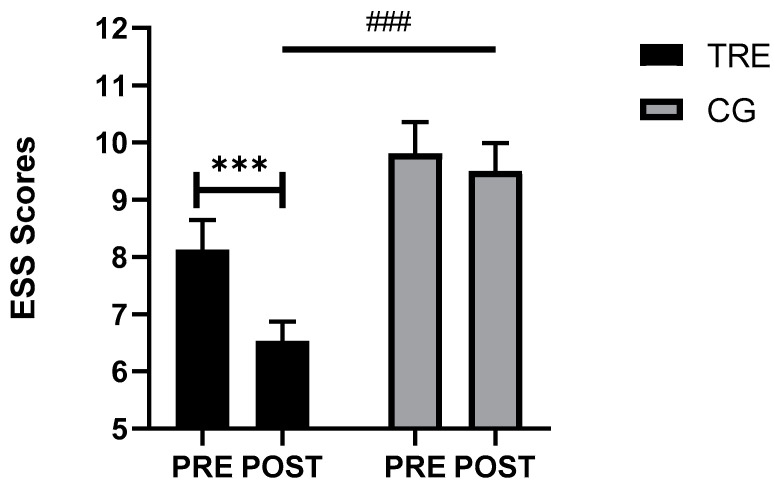
Comparative analysis of somnolence scale scores across the different groups. TRE: time-restricted eating; CG: control Group; PRE: before the intervention; POST: after the intervention. *** <0.0005, comparison between pre- and post-intervention; ### <0.0005, comparison between the groups in post intervention.

Table 2 presents the data on the sleep parameters before and after the intervention. The statistical analysis revealed non-significant changes across all the objectively measured parameters. However, there was a significant effect of time (F_(1, 29)_ = 12.61, *p* = 0.001, ηp^2^ = 0.30) group (F_(1, 29)_ = 14.30, *p* = 0.001, ηp^2^ = 0.33) and (time × group) interaction (F_(1, 29)_ = 5.72, *p* = 0.02, ηp^2^ = 0.02) for the ESS scores. The Bonferroni post hoc test showed that the scores were significantly lower in the TRE group after the intervention compared to before the intervention (*p* < 0.0005). Furthermore, during the post-intervention test, these scores were lower in the TRE compared to the CG group (*p* < 0.0005) (Figure 3).

### 3.4. Respiratory Function

**Figure 4:**
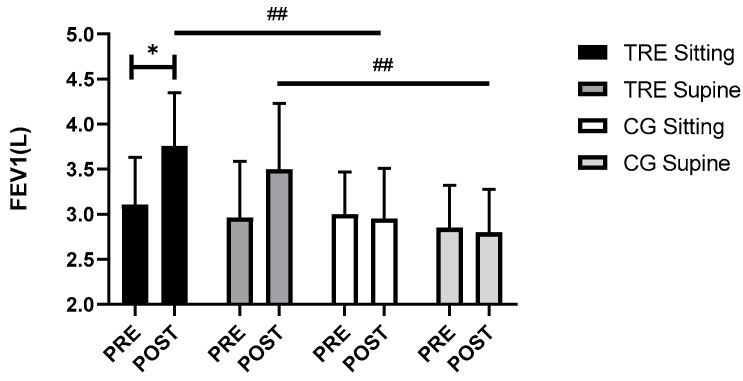
Variation in FEV1 across two positions among TRE and CG groups. FEV1: forced expiratory volume in the first second. TRE: time-restricted eating; CG: control group; PRE: before the intervention; POST: after the intervention; * *p* < 0.05, comparison between pre- and post-intervention; ## *p* < 0.01, comparison between groups in post intervention.

The spirometric parameters results are presented in Table 3. Three-way ANOVA revealed time (F_(1, 116)_ = 7.20, *p* = 0.008, ηp^2^ = 0.05), group (F_(1, 116)_ = 18.07, *p* < 0.0005, ηp^2^ = 0.13), and (time × group) interaction effects (F_(1, 116)_ = 10.03, *p* < 0.0005, ηp^2^ = 0.08) on FEV1. However, no significant group × position (*p* = 0.79) and group × position × time (*p* = 0.78) effects were observed. The Bonferroni post hoc analysis showed a significant increase in FEV1 in the TRE group during both sitting (*p* < 0.0005) and supine positions (*p* = 0.001) compared to the CG group. Higher FEV1 values in TRE were observed after the intervention in the sitting position (*p* = 0.03) (Figure 4). Nevertheless, there were no significant differences observed for the FVC and FEV1/FVC ratio, either within or between the groups.

### 3.5. Postural Balance 

The three-way ANOVA analysis revealed a significant effect of group in [A] (F(_1, 116)_ = 34.57, *p* < 0.0005, ηp^2^ = 0.23) and [PM] directions (F_(1, 116)_ = 5.39, *p* = 0.02, ηp^2^ = 0.04). Although, a significant effect of interaction (group × time) in [A] (F_(1, 116)_ = 7.03, *p* = 0.009, ηp^2^ = 0.05) and in [PM] (F_(1, 116)_ = 5.21, *p* = 0.02, ηp^2^ = 0.04) was observed. The Bonferroni post hoc test showed that performance in A (*p* = 0.03) and PM (*p* = 0.04) direction was higher in TRE after the intervention and compared to the CG (*p* < 0.0005, *p* = 0.001, respectively) with no difference between both legs in all three directions (Table 4). Although, performance in the PL direction was higher in the TRE group compared to the CG (*p* = 0.003).

### 3.6. Relationship between Body Composition and Physio-Mechanical Parameters

Only a significant negative correlation was observed between the fat mass and anterior direction postural performance (pre/post) delta changes among the TRE group participants (r = 0.553, *p* = 0.032). In the same group, a non-significant negative correlation trend was found between the (pre/post) delta changes of weight and FEV1 in the sitting position (r = −0.507, *p* = 0.054).

## 4. Discussion

The present study aimed to investigate the effect of time-restricted eating (TRE) without caloric restriction on heart rate variability (HRV), objective sleep, pulmonary function, and postural balance in overweight or obese women. The main findings of the study were as follows: After the intervention, no adverse effect of TRE on HRV and objective sleep assessments were observed when compared with the CG group. However, TRE induced an enhancement in subjective sleep parameters, FEV1, and postural balance in the anterior, postero-medial and postero-lateral positions. 

In the present study, the absence of changes in the HRV assessments, including mean RR, SDNN, LF, HF, HF/LF, and RMSSD agrees with the finding of Hammoud et al. [47] who did not observe change in RMSSD before and after four weeks of RIF. However, another study conducted by Mzoughi et al. [48] observed a decrease in HRV, characterized by lower SDNN, along with an enhancement of the sympathetic nervous system activity during fasting among hypertensive subjects, as compared to the period after RIF. On the other hand, the study by Cansel et al. [29] revealed an increase in HRV parameters during RIF with a fasting period of 18 h or more during the summer months. These discrepancies between findings regarding the interaction between fasting and autonomic nervous system responses can be elucidated by different factors such as individual age and health status [49]. Additionally, the timing of the measurements between fasting and feeding states may affect the results. Previous research suggested that in non-fasting conditions, HRV trends to decline from morning to evening; this decline can be attributed to continuous food ingestion throughout the day and the metabolic demands of the body [47]. That being said, further investigations are needed to describe the impact of the time of day on HRV assessments in chronic fasting conditions.

Numerous studies have delved into the analysis of sleep structure in relation to RIF, while relatively few studies have explored the specific effects of TRE on sleep architecture through objective assessments. The results from the present study indicate that TRE intervention exhibits no discernable negative impact on sleep, supported by consistent sleep duration, timing, and the enhancement of subjective Epworth scores for the same group over the twelve-week fasting period, aligning with previous findings in individuals with obesity, which indicated an absence of sleep disturbances induced by TRE [50]. Our findings suggest that the absence of negative effects on sleep patterns can be attributed to the favorable sleep quality exhibited by our cohort at the study’s initiation. The baseline mean sleep duration of eight hours among our participants aligns closely with the National Sleep Foundation’s recommended minimum of 7 h [51]. Conversely, Steger et al. [52] demonstrated that engaging in TRE might potentially result in adverse effects on sleep when coupled with an energy-restricted diet. The participants adhering to the TRE + food restriction reported taking a few additional minutes to fall asleep and experienced a reduction of half an hour in total sleep duration compared to the adherents in the control group [52]. A conceivable explanation for these observations is that adherents practicing TRE with calorie-restricted diet may have experienced increased nighttime hunger, potentially disrupting their sleep. This may explain the trend in increasing latency rates in our experimental group. However, it is important to note that the absence of negative effects on HRV and sleep in the present study may also be explained by the fasting duration and the chronobiological phenotype of the participants in TRE in our study and Ramadan fasting in previous research [48]. A more in-depth analysis is needed to thoroughly explore and understand these observed findings.

To the authors’ knowledge, this is the first study which evaluated the effect of TRE on spirometric assessments in sitting and supine positions in women with excess body weight. Our results did not indicate a statistically significant difference between the two positions. Nevertheless, FEV1 had more change from baseline (before fasting) and larger improvement in the TRE patients compared to the non-fasting ones, and this may show a potential positive effect of fasting on lung function in obese females. 

The observed beneficial impact of fasting on this parameter may be attributed to the weight loss experienced during fasting [53]. 

In a study conducted by Hakala et al. [54] involving fifteen overweight asthmatic patients, the implementation of a low-calorie diet resulted in weight loss, subsequently leading to a reduction in airway constriction. Moreover, some other mechanisms were previously recognized in some studies to be able to modify lung function and reduce disease severity during fasting. These enhancements can be attributed to the reduction in food allergens, decreased stomach volume resulting in reduced pressure on the diaphragm, and reduction in gastroesophageal reflux disease. 

The present study offers valuable insights into the relationship between TRE regimen and postural balance in overweight or obese women. The fasting intervention involving a 16 h period demonstrated an improvement in the participants’ ability to maintain balance, as assessed through the Y balance test in three directions and with both legs. Notably, previous studies have reported a decline in balance among functionally elite men [33] and the elderly people [34]. The decline in postural balance in these studies was explained by a change in lifestyle, meals, and activity schedules to chronobiology modification.

Up until now, only one study has investigated the effect of 48 h acute fasting on both static and dynamic balance in overweight or obese women [55]. According to these authors, an improvement in balance was noted when visual input was suppressed. This enhancement may be explained by enhanced proprioceptive and/or vestibular function [56]. In the same vein, the improvement in postural balance observed in the present study may be attributed to the consistent average sleep quality before and after the intervention. The participants in the TRE group were required to cease eating before eight am, thus avoiding sleep deprivation and ensuring no decline in sleep quality that could affect postural stability. 

Based on our findings, TRE fasting can be considered as a safe practice with the potential to enhance the health of higher-weight women. One of the principal limitations of the present pilot study was the absence of a non-fasting exercise group. Additionally, the dietary aspects, including calorie and macronutrient composition, were not monitored throughout the study. Furthermore, it is crucial to recognize that the impact of TRE may vary between males and females, underscoring the importance of acknowledging sex differences and conducting dedicated analyses in future research. Our conclusions should be regarded as a preliminary exploration. 

## 5. Conclusions

This study reveals notable improvements in FEV1, demonstrating enhancements in both sitting and supine positions following a 12-week period of TRE among overweight or obese women. The observed positive changes in FEV1 underscore the potential respiratory benefits associated with TRE, suggesting a favorable impact on pulmonary function. Additionally, the improved postural balance across various directions highlights the broader physiological implications of this dietary approach. It is essential to underscore that TRE did not have adverse effects on the sleep and HRV assessments. Specifically, there was no decline in sleep quality, and the duration of sleep remained unchanged. Further research is needed to investigate the correlation between the autonomic nervous system, pulmonary function, and balance regulation during training exercises in a fasted state. Understanding the implications of TRE on these crucial physiological aspects can provide valuable insights for optimizing health and performance strategies, especially in the context of weight management and metabolic health for individuals with overweight or obesity.

## Figures and Tables

**Table 1:** Participants characteristics.

Parameters	TRE		CG							
	PRE	POST	PRE	POST	F_(1, 29)_	*p* (T)	ηp^2^	F_(1, 29)_	*p* (T × G)	ηp^2^
**Weight (kg)**	88.31 ± 13.37	84.48 ± 13.37	90.87 ± 19.01	92.03 ± 19.50	13.53	0.001	0.31	46.97	<0.0005	0.61
**FM (kg)**	38.52 ± 5.37	37.75 ± 5.36	40.66 ± 7.14	40.99 ± 6.41	0.06	0.80	0.002	0.38	0.53	0.01
**FM (%)**	44.02 ± 5.71	45.25 ± 6.91	46.12 ± 11.07	45.97 ± 10.54	0.23	0.63	0.008	0.38	0.54	0.01
**FFM (kg)**	49.79 ± 11.29	46.73 ± 12.11	50.21 ± 19.17	51.03 ± 19.13	1.57	0.22	0.05	4.74	0.04	0.14
**Steps (nb)**	9388.1 ± 3422.9	9913.8 ± 3697.4	11,878.7 ± 1356.5	7619.9 ± 2192.3	0.92	0.34	0.03	1.52	0.22	0.05
**METs/h**	1.39 ± 0.34	1.61 ± 0.30	1.59 ± 0.22	1.25 ± 0.30	0.53	0.46	0.01	0.30	0.001	0.30
**EE (kcal)**	339.02 ± 174.18	457.26 ± 430.07	258.85 ± 75.24	255.21 ± 75.22	0.85	0.36	0.02	0.96	0.33	0.03

Values are means ± SD. TRE: time-restricted eating group; CG: control group; FM: fat mass; FFM: Fat-Free Mass; METs/h: resting metabolic equivalent of task; EE: energy expenditure; PRE: before the intervention; POST: after the intervention T: time effect; G: group effect; (T × G): time × group effect.

**Table 2:** Objective Sleep parameters across the intervention.

Parameters	TRE		CG		F	*p* (T)	ηp^2^	F	*p* (G)	ηp^2^	F	*p* (T × G)	ηp^2^
	PRE	POST	PRE	POST									
**Efficiency (%)**	94.80 + 2.77	94.64 + 2.47	95.04 + 1.88	94.39 + 3.21	0.41	0.52	0.01	0.1	0.99	0.004	0.15	0.70	0.005
**Latency (min)**	0.13 + 0.23	0.37 + 0.66	0.41 + 0.45	0.38 + 0.40	0.69	0.41	0.02	0.18	0.66	0.006	9.44	0.005	0.24
**WASO (min)**	25.28 + 18.11	26.66 + 10.91	24.17 + 6.64	21.67 + 14.19	0.02	0.87	0.001	0.90	0.35	0.03	0.31	0.57	0.01
**TIB (min)**	509.34 + 115.88	510.85 + 65.73	517.14 + 101.79	463.17 + 119.97	1.39	0.24	0.04	0.44	0.50	0.01	1.56	0.22	0.05
**TST (min)**	485.26 + 103.14	485.82 + 68.63	494.94 + 102.50	438.35 + 114.61	1.83	0.18	0.05	0.42	0.51	0.01	1.90	0.17	0.06

Values are means ± SD. TRE: time-restricted eating group; CG: control group; WASO: wake after sleep onset; TIB: time in bed; TST: total sleep time; PRE: before the intervention; POST: after the intervention T: time effect; G: group effect; (T × G): time × group effect.

**Table 3:** Comparison of spirometry parameters between groups in sitting and supine positions.

Parameters	TRE		CG		F (G × P)	*p* (G × P)	ηp^2^ (G × P)	F (G × T × P)	*p* (G × T × P)	ηp^2^ (G × T × P)
	PRE	POST	PRE	POST						
**FVC in sitting position (L)**	3.89 ± 0.67	3.76 ± 0.59	3.58 ± 0.53	3.61 ± 0.65	0.14	0.70	0.001	0.06	0.79	0.001
**FVC in supine position (L)**	3.66 ± 0.79	3.84 ± 0.38	3.49 ± 0.60	3.72 ± 0.45						
**FEV1 in sitting position (L)**	3.11 ± 0.52	3.76 ± 0.59	3.00 ± 0.47	2.95 ± 0.56	0.07	0.79	0.001	0.05	0.81	0.000
**FEV1 in supine position (L)**	2.96 ± 0.63	3.50 ± 0.73	2.85 ± 0.47	2.80 ± 0.48						
**FEV1/FVC in sitting position (%)**	82.02 ± 5.89	82.20 ± 5.19	82.57 ± 4.43	81.90 ± 5.32	0.49	0.48	0.007	0.18	0.67	0.004
**FEV1/FVC in supine position (%)**	79.16 ± 5.75	81.40 ± 5.81	81.85 ± 4.69	81.62 ± 4.88						

Values are means ± SD. TRE: time-restricted eating; CG: control group; FVC: forced vital capacity; FEV1: forced expiratory volume in the first second; PRE: before the intervention; POST: after the intervention T: time effect; G: group effect; P: position effect; G × P: group × position effect; G × T × P: group × time × position effect.

**Table 4:** Comparative analysis of postural balance performance in three distinct directions across different groups.

Parameters	TRE		CG		F	*p* (T)	ηp^2^	F	*p* (L)	ηp^2^	F	*p* (G × T × L)	ηp^2^
	PRE	POST	PRE	POST									
**Anterior R (cm)**	69.86 ± 6.87	73.4 ± 6.64	66.87 ± 3.73	64.18 ± 3.85	0.20	0.65	0.002	4.48	0.03	0.037	0.32	0.57	0.003
**Anterior L (cm)**	67.93 ± 6.45	64.68 ± 3.85	70.40 ± 6.18	63.12 ± 4.01									
**Postero-Medial R (cm)**	67.53 ± 6.68	70.46 ± 6.33	67.18 ± 7.64	65.12 ± 6.99	0.33	0.56	0.003	0.001	0.97	0.000	0.06	0.80	0.001
**Postero-Medial L (cm)**	67.00 ± 6.45	71.06 ± 5.68	67.25 ± 6.98	65.12 ± 7.39									
**Postero-Lateral R (cm)**	64.93 ± 6.70	67.86 ± 6.09	63.93 ± 6.86	63.00 ± 7.54	0.75	0.38	0.007	0.06	0.8	0.001	0.02	0.87	0.000
**Postero-Lateral L (cm)**	65.00 ± 6.08	68.40 ± 6.17	68.40 ± 6.17	63.12 ± 6.92									

Values are means ± SD. TRE: time-restricted eating; CG: control group; PRE: before the intervention; POST: after the intervention; T: time effect; G: group effect; G × T × L: group × time × leg effect; L: leg effect; R: right leg; L: left leg.

## Data Availability

The original contributions presented in the study are included in the article, further inquiries can be directed to the corresponding author.

## References

[1] Chang Y., Du T., Zhuang X., Ma G. (2024). Time-restricted eating improves health because of energy deficit and circadian rhythm: A systematic review and meta-analysis. iScience.

[2] Lopez-Jimenez F., Almahmeed W., Bays H., Cuevas A., Di Angelantonio E., le Roux C.W., Sattar N., Sun M.C., Wittert G., Pinto F.J. (2022). Obesity and cardiovascular disease: Mechanistic insights and management strategies. A joint position paper by the World Heart Federation and World Obesity Federation. Eur. Heart J..

[3] Patel M., Braun J., Lambert G., Kameneva T., Keatch C., Lambert E. (2023). Central mechanisms in sympathetic nervous dysregulation in obesity. J. Neurophysiol..

[4] Laederach-Hofmann K., Mussgay L., Rúddel H. (2000). Autonomic cardiovascular regulation in obesity. J. Endocrinol..

[5] Espinoza-Salinas A., Peiret-Villacura L., Molina-Sotomayor E., Cigarroa-Cuevas I., Arenas-Sánchez G., Podestá I., González-Jurado J. (2022). Effects of Cardiovagal Training on Autonomic Function, Inflammatory Markers and Insulin Levels in Adults with Obesity. Preprints.

[6] Shah N.M., Kaltsakas G. (2023). Respiratory complications of obesity: From early changes to respiratory failure. Breathe.

[7] Grassi L., Kacmarek R., Berra L. (2020). Ventilatory Mechanics in the Patient with Obesity. Anesthesiology.

[8] Lo Mauro A., Tringali G., Codecasa F., Abbruzzese L., Sartorio A., Aliverti A. (2023). Pulmonary and chest wall function in obese adults. Sci. Rep..

[9] Zhou N., Forton K., Motoji Y., Scoubeau C., Klass M., Naeije R., Faoro V. (2022). Right ventricular-pulmonary arterial coupling impairment and exercise capacity in obese adults. Front. Cardiovasc. Med..

[10] Nanayakkara B., McNamara S. (2024). Pathophysiology of Chronic Hypercapnic Respiratory Failure. Sleep. Med. Clin..

[11] Hagenburg J., Bertin E., Salmon J.-H., Thierry A., Perotin J.-M., Dormoy V., Dury S., Gaubil I., Bolko L., Lebargy F. (2022). Association between obesity-related dyspnea in daily living, lung function and body composition analyzed by DXA: A prospective study of 130 patients. BMC Pulm. Med..

[12] Oppert J.-M., Ciangura C., Bellicha A. (2023). Physical activity and exercise for weight loss and maintenance in people living with obesity. Rev. Endocr. Metab. Disord..

[13] Rijal A., Adhikari T.B., Dhakal S., Maagaard M., Piri R., Nielsen E.E., Neupane D., Jakobsen J.C., Olsen M.H. (2024). Effect of exercise on functional capacity and body weight for people with hypertension, type 2 diabetes, or cardiovascular disease: A systematic review with meta-analysis and trial sequential analysis. BMC Sports Sci. Med. Rehabil..

[14] Verdú E., Homs J., Boadas-Vaello P. (2021). Physiological Changes and Pathological Pain Associated with Sedentary Lifestyle-Induced Body Systems Fat Accumulation and Their Modulation by Physical Exercise. Int. J. Environ. Res. Public Health.

[15] Tutor A.W., Lavie C.J., Kachur S., Milani R.V., Ventura H.O. (2023). Updates on obesity and the obesity paradox in cardiovascular diseases. Prog. Cardiovasc. Dis..

[16] Tashiro H., Kurihara Y., Kuwahara Y., Takahashi K. (2024). Impact of obesity in asthma: Possible future therapies. Allergol. Int..

[17] Peters U., Dixon A.E. (2018). The effect of obesity on lung function. Expert Rev. Respir. Med..

[18] Al Lawati R., Al Abri M.A., Kuppuswamy B., Al-Kharousi A., Al-Atbi A.Y., Rizvi S., Dikshit M. (2019). The Effect of Change in Posture on Spirometry in Patients with Obstructive Sleep Apnoea Syndrome. Sultan Qaboos Univ. Med. J..

[19] Antza C., Kostopoulos G., Mostafa S., Nirantharakumar K., Tahrani A. (2022). The links between sleep duration, obesity and type 2 diabetes mellitus. J. Endocrinol..

[20] Zhou Y., Guo X., Liu Z., Sun D., Liang Y., Shen H., Li X., Mu J., Liu J., Cao G. (2024). 6-week time-restricted eating improves body composition, maintains exercise performance, without exacerbating eating disorder in female DanceSport dancers. J. Int. Soc. Sports Nutr..

[21] Manoogian E.N.C., Chow L.S., Taub P.R., Laferrère B., Panda S. (2022). Time-restricted Eating for the Prevention and Management of Metabolic Diseases. Endocr. Rev..

[22] Crose A., Alvear A., Singroy S., Wang Q., Manoogian E., Panda S., Mashek D.G., Chow L.S. (2022). Time-Restricted Eating Improves Quality of Life Measures in Overweight Humans. Nutr. J..

[23] Jamshed H., Beyl R.A., Della Manna D.L., Yang E.S., Ravussin E., Peterson C.M. (2019). Early Time-Restricted Feeding Improves 24-Hour Glucose Levels and Affects Markers of the Circadian Clock, Aging, and Autophagy in Humans. Nutr. J..

[24] Wang R., Liao Y., Deng Y., Shuang R. (2024). Unraveling the Health Benefits and Mechanisms of Time-Restricted Feeding: Beyond Caloric Restriction. Nutr. Rev..

[25] Mackieh R., Al-Bakkar N., Kfoury M., Okdeh N., Pietra H., Roufayel R., Legros C., Fajloun Z., Sabatier J.-M. (2023). Unlocking the Benefits of Fasting: A Review of Its Impact on Various Biological Systems and Human Health. Curr. Med. Chem..

[26] Lin Y.-J., Wang Y.-T., Chan L.-C., Chu N.-F. (2022). Effect of time-restricted feeding on body composition and cardio-metabolic risk in middle-aged women in Taiwan. Nutrition.

[27] Zimmermann P., Herz D., Karl S., Weiß J.W., Lackner H.K., Erlmann M.P., Sourij H., Schierbauer J., Haupt S., Aberer F. (2023). Effects of Different Fasting Interventions on Cardiac Autonomic Modulation in Healthy Individuals: A Secondary Outcome Analysis of the EDIF Trial. Biology.

[28] Kammoun N., Hidouri S., Ghram A., Ammar A., Masmoudi L., Driss T., Knechtle B., Weiss K., Hammouda O., Chlif M. (2022). Effects of Walking Football during Ramadan Fasting on Heart Rate Variability and Physical Fitness in Healthy Middle-Aged Males. Am. J. Mens. Health.

[29] Cansel M., Taşolar H., Yağmur J., Ermiş N., Açıkgöz N., Eyyüpkoca F., Pekdemir H., Ozdemir R. (2014). The effects of Ramadan fasting on heart rate variability in healthy individuals: A prospective study. Anadolu Kardiyol. Derg..

[30] Sibley K.M., Mochizuki G., Lakhani B., McIlroy W.E. (2014). Autonomic contributions in postural control: A review of the evidence. Rev. Neurosci..

[31] Hall W.L. (2022). The emerging importance of tackling sleep–diet interactions in lifestyle interventions for weight management. Br. J. Nutr..

[32] Cay M., Ucar C., Senol D., Cevirgen F., Ozbag D., Altay Z., Yildiz S. (2018). Effect of increase in cortisol level due to stress in healthy young individuals on dynamic and static balance scores. North. Clin. Istanb..

[33] Souissi N., Zouita A., Chtourou H., Ferchichi H., Dziri C., Abedelmalek S., Souissi N. (2014). The effect of Ramadan intermittent fasting on dynamic postural control in judo athletes. Biol. Rhythm. Res..

[34] Laatar R., Borji R., Baccouch R., Zahaf F., Rebai H., Sahli S. (2016). Effects of Ramadan Fasting on Postural Balance and Attentional Capacities in Elderly People. J. Nutr. Health Aging.

[35] Izadi M., Thomas E., Thomas A.C., Bellafiore M. (2022). The effect of time-of-day and sleep deprivation on postural control: A systematic review. Gait Posture.

[36] Paillard T. (2023). Detrimental effects of sleep deprivation on the regulatory mechanisms of postural balance: A comprehensive review. Front. Hum. Neurosci..

[37] Kamoun A., Hammouda O., Yahia A., Dhari O., Ksentini H., Driss T., Souissi N., Elleuch M.H. (2019). Effects of Melatonin Ingestion Before Nocturnal Sleep on Postural Balance and Subjective Sleep Quality in Older Adults. J. Aging Phys. Act..

[38] Serdar C.C., Cihan M., Yücel D., Serdar M.A. (2021). Sample size, power and effect size revisited: Simplified and practical approaches in pre-clinical, clinical and laboratory studies. Biochem. Med..

[39] Alamer R., Khalifa K., Alajlan S., Al Ansari A. (2018). Analyzing the Psychometric Properties of the Short Form-36 Quality of Life Questionnaire in Patients with Obesity. Obes. Surg..

[40] Xie X., Zhang M., Luo H. (2024). Regulation of metabolism by circadian rhythms: Support from time-restricted eating, intestinal microbiota & omics analysis. Life Sci..

[41] Moro T., Tinsley G., Bianco A., Marcolin G., Pacelli Q.F., Battaglia G., Palma A., Gentil P., Neri M., Paoli A. (2016). Effects of eight weeks of time-restricted feeding (16/8) on basal metabolism, maximal strength, body composition, inflammation, and cardiovascular risk factors in resistance-trained males. J. Transl. Med..

[42] Martin J.L., Hakim A.D. (2011). Wrist actigraphy. Chest.

[43] Tinsley G.M., Moore M.L., Graybeal A.J., Paoli A., Kim Y., Gonzales J.U., Harry J.R., VanDusseldorp T.A., Kennedy D.N., Cruz M.R. (2019). Time-restricted feeding plus resistance training in active females: A randomized trial. Am. J. Clin. Nutr..

[44] Miller M.R., Pedersen O.F., Dirksen A. (2007). A new staging strategy for chronic obstructive pulmonary disease. Int. J. Chron. Obs. Pulmon Dis..

[45] Suvarna T., Oliver Raj J., Prakash N. (2021). Correlation between Balance and BMI in Collegiate students: A cross sectional study. Int. J. Physiother. Res..

[46] Lakens D. (2013). Calculating and reporting effect sizes to facilitate cumulative science: A practical primer for t-tests and ANOVAs. Front. Psychol..

[47] Hammoud S., Mourad R., Karam R., Saad I., van den Bemt B.J.F., Kurdi M. (2020). Effect of Ramadan fasting on heart rate variability as a measure of cardiac stress in a Lebanese cohort. Eur. J. Clin. Nutr..

[48] Mzoughi K., Zairi I., Jabeur M., Kraiem S. (2018). The effects of fasting on heart rate variability in hypertensive patients. Clin. Exp. Hypertens..

[49] Rodrigues J.A.L., Yamane A.C., Gonçalves T.C.P., Kalva-Filho C., Papoti M., Júnior C.R.B. (2019). Fed and fasted states on heart rate variability, hemodynamic heart rate and blood pressure in adults submited to moderate aerobic exercise. Int. J. Cardiol. Heart Vasc..

[50] Gabel K., Hoddy K.K., Varady K.A. (2019). Safety of 8-h time restricted feeding in adults with obesity. Appl. Physiol. Nutr. Metab..

[51] Metse A.P., Bowman J. (2020). A Prevalence of self-reported suboptimal sleep in Australia and receipt of sleep care. Sleep. Health.

[52] Steger F.L., Jamshed H., Bryan D.R., Richman J.S., Warriner A.H., Hanick C.J., Martin C.K., Salvy S.-J., Peterson C.M. (2023). Early time-restricted eating affects weight, metabolic health, mood, and sleep in adherent completers: A secondary analysis. Obesity.

[53] Latiri I., Sandid S., Fennani M.A., Hadrich M., Masmoudi T., Maatoug C., Zammit-Chatti M., Chamari K., Ben Saad H. (2017). The Effects of Ramadan Fasting on the Spirometric Data of Healthy Adult Males. Am. J. Mens. Health.

[54] Hakala K., Stenius-Aarniala B., Sovijärvi A. (2000). Effects of weight loss on peak flow variability, airways obstruction, and lung volumes in obese patients with asthma. Chest.

[55] Solianik R., Žlibinaitė L., Drozdova-Statkevičienė M., Sujeta A. (2020). Forty-eight-hour fasting declines mental flexibility but improves balance in overweight and obese older women. Physiol. Behav..

[56] Redfern M.S., Yardley L., Bronstein A.M. (2001). Visual influences on balance. J. Anxiety Disord..

